# Can Facebook Reduce Perceived Anxiety Among College Students? Randomized Controlled Exercise Trial Using the Transtheoretical Model of Behavior Change

**DOI:** 10.2196/mental.8086

**Published:** 2017-12-08

**Authors:** Emily Frith, Paul Loprinzi

**Affiliations:** ^1^ University of Mississippi University, MS United States

**Keywords:** mental health, physical activity behavior change, social media

## Abstract

**Background:**

Recent studies suggest social media may be an attractive strategy to promote mental health and wellness. There remains a need to examine the utility for individually tailored wellness messages posted to social media sites such as Facebook to facilitate positive psychological outcomes.

**Objective:**

Our aim was to extend the growing body of evidence supporting the potential for social media to enhance mental health. We evaluated the influence of an 8-week social media intervention on anxiety in college students and examined the impact of dynamic (active) versus static (passive) Facebook content on physical activity behaviors.

**Methods:**

Participants in the static group (n=21) accessed a Facebook page featuring 96 statuses. Statuses were intended to engage cognitive processes followed by behavioral processes of change per the transtheoretical model of behavior change. Content posted on the static Facebook page was identical to the dynamic page; however, the static group viewed all 96 statuses on the first day of the study, while the dynamic group received only 1 to 2 of these status updates per day throughout the intervention. Anxiety was measured using the Overall Anxiety Severity and Impairment Scale (OASIS). Time spent engaging in physical activity was assessed using the International Physical Activity Questionnaire (IPAQ).

**Results:**

The OASIS change score for the dynamic Facebook group was statistically significant (*P*=.003), whereas the change score for the static group was not (*P*=.48). A statistically significant group-by-time interaction was observed (*P*=.03). The total IPAQ group-by-time interaction was not statistically significant (*P*=.06).

**Conclusions:**

We observed a decrease in anxiety and increase in total physical activity for the dynamic group only. Dynamic social networking sites, featuring regularly updated content, may be more advantageous than websites that retain static content over time.

**Trial Registration:**

ClinicalTrials.gov NCT03363737; https://clinicaltrials.gov/ct2/show/NCT03363737 (Archived by WebCite at http://www.webcitation.org/6vXzNbOWJ)

## Introduction

Verduyn and colleagues [1] suggest a passive online presence on social networking sites is detrimental to mental health. A passive presence involves 1-sided consumption of digital media, devoid of attempts to connect with fellow users [2]. Passive social networking site behaviors may encourage unhealthy comparisons and jealousy, which may exert profoundly negative effects on psychological health. Conversely, an active social presence may predict perceived well-being via intentional connectivity, which serves to expand and strengthen social capital among social networking site members [1,2]. Kreps and Neuhauser [3] support the progressive social relevancy of online communication technologies to promote health, where viewable content is interactive and user-centered. To this end, target audiences are given the power to embark upon a meaningful health journey capable of addressing their unique goals and concerns [3]. The digital age has influenced widespread transmission of relational information. Tailored wellness messages posted to social media sites such as Facebook access specific populations who receive instantaneous virtual feedback, which may help to facilitate social support, physical health, and psychological well-being [1,4,5]. However, despite recent strides in mobile health (mHealth) research, there remains a need to further parse the significance of active versus passive mHealth interventions on mediating psychological outcomes among social networking site users. This pilot study evaluates the relationship between a Facebook-based exercise intervention and level of anxiety among college students, as anxiety is both a widespread and underdiagnosed public health concern within this population [6].

Although mHealth is less investigated than traditional, face-to-face therapeutic interventions, emerging work has begun to evaluate the effectiveness of online-based mHealth interventions [7]. The growing body of existing social media research lacks adherence to validated theoretical models and evidence-based practice [8,9]. Additionally, many websites contain content which fails to elicit sustained health behavior change [10]. Social media offers researchers the opportunity to assume an active and dynamic online presence, posting relevant wellness content, which, ideally, could increase user interaction and dissemination of promoted health topics. Recent interventions have illuminated the potential for social networking sites to function not only as popular communication platforms but also provide reliable hubs for accessing health information [11,12]. Social networking site and mobile phone apps are inexpensive, private, collaborative, and offer opportunities for rapid communication between users within an expansive social context, making virtual content attractive to young men and women around the globe [13,14].

Collectively, recent studies have demonstrated social media may be an attractive strategy to promote mental health and wellness. Social media champions a robust individual social presence, as well as mass interactivity, which may contribute to user satisfaction and subjective well-being [1]. These constituents enable users within social networking site communities to cultivate relationships and disseminate loadable content, which may begin as individually generated information but propagate virally as active users share media relevant to the generation of popular culture. Facebook use among college students is influenced most heavily by opportunities for members to develop a collaborative social presence [15]. Social support has been shown to facilitate enhanced perceptions of personal well-being and fulfillment [1,16,17]. Social media has the power to evoke feelings of belonging and, perhaps, raise self-esteem within users [18]. Such interactive content mobilization offers unique educational opportunities and may be a springboard to link public engagement with the wellness domain.

Firth et al [7] conducted a meta-analysis of 9 randomized controlled trials that evaluated the utility for mHealth interventions to favorably influence anxiety symptoms. The authors suggest the potential for effective digital technology strategies to significantly reduce anxiety. Among 4 reviewed interventions that specifically targeted anxiety outcomes, 2 demonstrated no reductions in anxiety beyond those observed in waitlist control groups [19,20]. Notably, 2 interventions reduced anxiety significantly more than waitlist conditions but were not superior to active controls [21,22]. Therefore, the magnitude of efficacy for mobile phone research remains unclear [23], and thus, requires additional research. In comparison to the research in Firth’s meta-analysis, we used the transtheoretical model of health behavior change rather than employing strategies specifically designed to treat mental disorders because this model has been shown to be associated with reduced anxiety symptoms [23]. Further, the transtheoretical model is widely used in physical activity research, and exercise is known to exert beneficial effects on mood profile, particularly within the college-aged population [23-25]. Additionally, unlike the studies included in the aforementioned meta-analysis, we did not use indication of mental health issues as inclusionary criteria and recruited from the general student body in an attempt to comprehensively address the health needs of the average college adult, who may be reluctant to expose their mental health concerns upon recruitment [26,27]. To this end, our pilot study aims to extend the growing body of evidence supporting the use of mobile phones as tools to promote mental health, address important gaps in the literature, and provide direction for future interventions evaluating the link between mobile phones and mental health. The specific purpose of this brief report was to examine the utility of an 8-week Facebook-based wellness intervention to serve as a platform for researchers to promote reliable and accurate wellness information. The primary objectives of the study were 2-fold: we aimed to examine the potential for dynamic (active) versus static (passive) Facebook content to influence physical activity behavior and evaluate the influence of a social media intervention on anxiety within a sample of undergraduate and graduate college students. Such a side-by-side comparison between an active versus static media-based intervention on physical activity is, to our knowledge, the first to be conducted.

## Methods

### Setting and Participants

**Figure 1:**
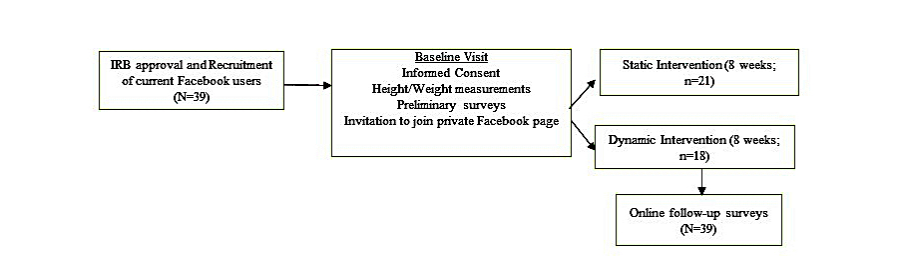
Study design schematic.

Undergraduate and graduate students (N=39; n=21 for static arm and n=18 for dynamic arm; see Figure 1) were recruited via convenience-based sampling at the University of Mississippi. Participants attended a baseline laboratory visit during which they provided consent, had their height and weight measured, completed preliminary surveys, and were given instructions to accept a researcher invitation to join a private Facebook page.

### Pilot Intervention

This pilot study was approved by the institutional review board at the University of Mississippi. Participants randomly allocated to join the static Facebook page (n=21) were exposed to a page that already contained 96 status updates targeting the precontemplation, contemplation, preparation, action, and maintenance phases of the transtheoretical model. The statuses progressed through general information tailored to participants’ community and university resources for healthy eating, stress and time management, physical activity benefits, and tips to implement positive lifestyle changes. Statuses were intended to offer participants awareness and specific tools necessary to engage cognitive processes of change (ie, consciousness raising, dramatic relief, self-evaluation, environmental evaluation, and social liberation) followed by behavioral processes of change (helping relationships, self-liberation, reinforcement, stimulus control, and counterconditioning) throughout the 8-week intervention period. Posts on the static Facebook page consisted of identical content as the dynamic group. The only distinction was that the static group received access to all 96 statuses on the first day of the study, while the dynamic group was exposed to 1 to 2 of these status updates per day across the 8-week intervention period.

Participants randomly allocated to join the dynamic Facebook page (n=18) were exposed to 2 status updates per weekday and 1 status update per weekend day for 7 weeks (96 status updates). During the final week, participants did not receive daily notifications to read status updates but were encouraged to complete online follow-up surveys. No incentives were provided to participants upon completion of this study.

### Main Outcome Measures

The primary outcomes assessed were changes in physical activity behavior and anxiety, measured via self-report. After meeting inclusionary criteria, which included having an active Facebook account, daily access to a mobile phone, and a current University of Mississippi email account, eligible participants were sent a Facebook invitation to join either the static or dynamic Facebook group. Time spent engaging in vigorous, moderate, and walking physical activity habits was assessed using the International Physical Activity Questionnaire (IPAQ), which has demonstrated adequate reliability and validity [28]. We created a total physical activity variable by summing the minutes per week of walking, moderate-intensity, and vigorous-intensity physical activity.

Anxiety was measured using the Overall Anxiety Severity and Impairment Scale (OASIS), a 5-item psychometric instrument completed via self-reported response on a 5-point Likert scale. A sample question from the OASIS is “How much does anxiety or fear interfere with your social life and relationships?” Higher ratings indicate greater anxiety inference. This scale has been used as a valid and reliable assessment of degree of anxiety impairment in various clinical and subclinical populations [29,30]. Internal consistency was calculated using Cronbach alpha for both the static (pre: .86 and post: .76) and dynamic group (pre: .72 and post: .84).

### Statistical Analyses

Stata SE version 12 (StataCorp LLC) was used to conduct paired sample *t* tests to examine change scores between pre- and post-OASIS scores for each group. An independent samples *t* test was then used to compare the change scores between the 2 intervention arms. For all analyses, statistical significance was established as a nominal alpha of .05.

## Results

Demographic information is displayed in Table 1. The OASIS change score for the static Facebook group was nonsignificant (*P*=.48). The static group exhibited a baseline mean score of 4.14 and a follow-up mean score of 3.57 on the OASIS. Conversely, the OASIS change score for the dynamic Facebook group was statistically significant (*P*=.003). The dynamic group exhibited a baseline mean score of 4.91 and a follow-up mean score of 1.36 on the OASIS. Change scores were then compared across both groups using an independent samples *t* test. A statistically significant group-by-time interaction was observed (*P*=.03).

**Table 1:** Demographic information.

Variable	Entire sample mean (SD)	Dynamic group mean (95% CI)	Static group mean (95% CI)
GPA^a^, baseline	3.26 (0.63)	3.31 (3.01 to 3.62)	3.05 (2.79 to 3.31)
GPA, postintervention	3.55 (0.46)	3.52 (3.17 to 3.87)	3.47 (3.14 to 3.81)
Age, years	22.65 (3.16)	21.9 (20.42 to 23.46)	22.2 (21.22 to 23.18)
Gender (% female)	68	75	81
Race (% white)	66	75	57
Years active on Facebook	7.97 (2.99)	7.56 (6.55 to 8.58)	8.62 (6.98 to 10.25)
Number of Facebook friends	1256 (949.92)	939.93 (558.47 to 1321.4)	1390.57 (1050.1 to 1731.04)
Number of logins per day, baseline	7.72 (6.2)	6.31 (4.92 to 7.71)	6.57 (4.32 to 8.82)
Number of logins per day, postintervention	4.35 (3.98)	3.73 (2.36 to 5.09)	4 (1.7 to 6.3)
Number of intervention-page logins per day	1.38 (0.58)	1.45 (1.12 to 1.79)	1.43 (0.8 to 2.06)
Total ambulatory physical activity, minutes per week	762.8 (1375.54)	(mean change score) 1378.25 (–25.61 to 2782.11)	(mean change score) 59.44 (–132.34 to 251.22)

^a^GPA: grade point average.

We used *t* tests to compare IPAQ pre- and postintervention change scores for physical activity behaviors between groups. The total IPAQ group-by-time interaction was not statistically significant (*P*=.06). We also computed the Mann-Whitney U analysis, which was not statistically significant (*P*=.13). Average reported total ambulatory movement (ie, light to vigorous physical activity) across groups was 762.8 minutes per week with a standard deviation of 1375.54. Mean ambulatory physical activity for the static group was 453.3 minutes per week at baseline and 540.72 minutes per week at follow-up. The average total physical activity change for this group was 59.44 minutes per week. Mean physical activity for the dynamic group was 366.62 minutes per week at baseline and 1393.1 minutes per week at follow-up. The average total physical activity change for the dynamic group was 1378.25 minutes per week. There was an inverse statistical correlation between changes in physical activity and anxiety. The zero-order correlation coefficient between mean physical activity change on mean anxiety change is *r*=–0.51 (*P*=.01).

## Discussion

### Principal Findings

The dearth of research examining the link between modern media consumption and anxiety is equivocal [31]. The relationship between mobile phone use and anxiety has been demonstrated among college students exhibiting compulsive or addictive mobile phone behaviors [32,33]. Lepp et al [31] suggest that investigations should attempt to further examine whether this correlation manifests for those not engaging in problematic usage of digital technology. We did not explicitly measure Internet addiction in this pilot intervention. However, our results indicate that for those allocated to the dynamic Facebook page, self-reported anxiety levels diminished significantly. Further, average general Facebook logins per day were marginally decreased for both groups following the intervention (see Table 1), suggesting our investigation did not contribute to addictive social networking behaviors.

Regarding the main outcomes, we observed a statistically significant decrease in anxiety for the dynamic group only. This finding may be influenced by our adherence to the transtheoretical model of behavior change. All status updates followed a theoretically driven sequence of messages in alignment with the cognitive and behavioral processes of change. Participants in the dynamic group received daily status updates containing accurate and credible health information. A total of 96 wellness messages were posted over 7 weeks with content first focusing on cognitive processes of change (ie, consciousness raising, dramatic relief, self-evaluation, environmental evaluation, and social liberation) followed by behavioral processes of change (helping relationships, self-liberation, reinforcement, stimulus control, and counterconditioning). Participants randomized into the static, or passive, Facebook page received access to all 96 messages on the first day of the intervention with no daily updates occurring thereafter. We hypothesize the potency of the cognitive and behavioral messages was diluted in the static group, providing rationale for the observed reduction in anxiety in the dynamic group alone. We also found a statistically significant inverse correlation between mean total physical activity change and mean change in anxiety. We feel this finding underscores the importance of daily, dynamic exposure to Facebook wellness messages. Physical activity behavior changes are difficult to sustain over time, so perhaps consistent social media notifications were advantageous reminders for participants in the dynamic group to increase total ambulatory movement throughout the 8-week intervention period. Thus, our findings suggest that dynamic social networking sites featuring regularly updated content may be more advantageous than websites that retain static content over time.

### Strengths and Limitations

Despite the plausibility for social media to be used effectively in physical and mental health research, there are numerous concerns that must be addressed when implementing social networking site interventions. Social media is user-centered, meaning individuals consume and produce content relevant to their unique goals and interests. Thus, efficacious social networking site research must be individually tailored. Lack of tailored content is a possible limitation of this pilot study. Although we observed increased changes in physical activity in the dynamic group, the majority of our recruited participants were exercise science or health promotion students already engaging in physical activity. Such extensive exposure to information specifically targeting initiation of positive health behaviors may have been less impactful in this sample. Cognitive and behavioral processes of change may be more appropriately tailored to individuals contemplating behavior change. Further, the risk of psychological disorders such as depression and anxiety is highest among young adults [34]. Importantly, this population is also unlikely to seek treatment, partially due to the stigmatization of mental health [26,27]. We were also unable to control for participant exposure to university courses, exercise programs, or access to wellness information outside of the pilot intervention, which may have affected physical activity or level of anxiety across the 8-week intervention period. A strength of this pilot study, as well as previous social networking site interventions, is the ability to directly target young adults, as social media use among millennials is unrivaled in today’s society [35]. However, social media inherently limits privacy, as it exists as a global platform designed to proliferate opportunities for social inclusion. Therefore, special consideration should be given to the social networking site outlet selected and precautionary measures taken to protect participant safety and anonymity. Our protocol addressed this issue appropriately by using a private, invitation-only Facebook page, although study participants were aware of fellow participants who had access to the page. The inability to completely blind participants may have limited participant interaction and comfortability in this study. Future research should design empirically based methodology that controls for participant privacy while retaining the social aspect integral to social media research.

### Conclusion

The results of this pilot study suggest reduced anxiety among college students may be associated with exposure to dynamic, or active, Facebook wellness interventions. We propose mHealth interventions be used as self-monitoring aids rather than for mental health diagnoses. That is, mobile phone technologies should not replace face-to-face therapeutic interventions but may be complementary to clinical services. Awareness and management of psychological health status may be improved through social media, which has been suggested to enhance self-efficacy and decrease the functional impairment associated with anxiety, depression, stress, and psychological disturbances [36]. Social media research is an emerging field deserving special investigation in university settings. Communication hubs including Facebook, Twitter, Instagram, Snapchat, and YouTube are powerful modern research tools. These sites offer the novel potential for rapid dissemination of tailored messages, which may revolutionize the field of psychological and behavioral health promotion.

## Abbreviations

IPAQ: International Physical Activity Questionnaire
OASIS: Overall Anxiety Severity and Impairment Scale
